# Assessing modifiable risk factors for dementia in the Czech Republic: findings from the Survey of Health, Ageing and Retirement in Europe study

**DOI:** 10.1093/eurpub/ckaf112

**Published:** 2025-07-09

**Authors:** Matej Kucera, Dominika Seblova, Judith E Bosmans, Hana Marie Broulikova, Pavla Brennan Kearns

**Affiliations:** Second Faculty of Medicine, Charles University, Prague, Czech Republic; Department of Health Sciences, Faculty of Science, Amsterdam Public Health Research Institute, Vrije Universiteit Amsterdam, Amsterdam, The Netherlands; Health Economics, Policy and Innovation Institute, Faculty of Economics and Administration, Masaryk University, Brno, Czech Republic; Second Faculty of Medicine, Charles University, Prague, Czech Republic; Department of Health Sciences, Faculty of Science, Amsterdam Public Health Research Institute, Vrije Universiteit Amsterdam, Amsterdam, The Netherlands; Department of Health Sciences, Faculty of Science, Amsterdam Public Health Research Institute, Vrije Universiteit Amsterdam, Amsterdam, The Netherlands; Public Mental Health Department, National Institute of Mental Health, Klecany, Czech Republic; Second Faculty of Medicine, Charles University, Prague, Czech Republic

## Abstract

The role of modifiable risk factors in the development of dementia in Central and Eastern Europe remains understudied. We aimed to examine the association between 12 risk factors and the incidence of dementia in the Czech Republic and estimate the proportion of new dementia cases that can be attributed to these risk factors. Data of 3805 Czech participants in the Survey of Health, Ageing, and Retirement in Europe (mean age: 70 years, median 6.5-year follow-up) were analyzed. Hazard ratios (HRs) with 95% confidence intervals (CIs) were estimated using Cox hazard models for the association between the risk factors (low education, alcohol use, living alone, obesity, smoking, physical inactivity, high blood pressure, high cholesterol, diabetes mellitus, hearing loss, vision problem, and depression) and probable dementia diagnosis defined based on adapted Lang-Weir algorithm. We estimated the proportion of dementia cases attributable to each risk factor using weighted population attributable fractions (wPAFs). Four risk factors, low education (HR 1.72), depression (HR 1.42), diabetes mellitus (HR 1.53), and physical inactivity (HR 2.13), were significantly associated with dementia and accounted for the largest proportion of attributable risk. The total weighted PAF for all factors was 39.18%. If all risk factors for dementia were eliminated, almost 40% of dementia cases in the Czech Republic could be prevented. More systematic approach is essential for mitigating the adverse impact of risk factors on the incidence of dementia, such as improving education, preventing and treating depression and diabetes mellitus, and promoting physical.

## Introduction

In 2024, the Lancet Commission published a life course model encompassing 14 potentially modifiable risk factors for dementia: low education, high blood pressure, hearing loss, smoking, obesity, depression, physical inactivity, diabetes, living alone, alcohol use, traumatic brain injury, vision problem, high cholesterol, and air pollution [1]. Collectively, these modifiable risk factors are estimated to account for approximately 45% of dementia cases. However, the level and quality of evidence used for this model varies across different regions. For example, none of the studies included in the meta-analysis was conducted in Central and Eastern Europe (CEE). The lack of sufficient epidemiological evidence in this region was also mentioned by a 2021 systematic review of population-based studies on risk and protective factors for neurocognitive disorders [2].

It is important to emphasize that many of the risk factors for neurocognitive disorders are highly prevalent in CEE countries, due to combination of several reasons such as socioeconomical factors, genetic predisposition, healthcare system, and dietary habits. In the Czech Republic, high blood pressure affects approximately one quarter of the population [3], with large treatment gaps observed [4]. One quarter of the population also reports being active smokers [5]. In addition, one quarter of males report daily or near-daily alcohol consumption [6]. Almost 20% of Czechs are overweight or obese [4]. One tenth has diabetes mellitus [7]. Depression is also prevalent and highly untreated, affecting more than 6% of the population [8], and this rate tripled during the Covid-19 pandemic [9]. Data from 2020 also revealed that approximately one-third of the Czech population fails to attain sufficient levels of physical activity [10].

Considering the high prevalence of known dementia risk factors in the Czech Republic, evidence on the contribution of these factors to the incidence of new cases of dementia can support decision makers in the implementation of interventions to prevent dementia. Such research is essential for developing effective evidence-based policies, aligning with the objectives of the Czech National Action Plan for Alzheimer's Disease and Related Illnesses 2020–2030. Capitalizing on a nation-wide population-based cohort, the aim of this study is to investigate the role of risk factors highlighted by Lancet Commission in the development of dementia in the Czech Republic.

## Methods

### Data source

The analysis was based on data from the Survey of Health, Ageing and Retirement in Europe (SHARE), which is a multidisciplinary, cross-national study that collects information about health, social network, and economic characteristics of the aging population in 28 European countries and Israel. The first wave of data was collected in 2004 and was followed by seven subsequent waves in 2006/2007 (wave 2), 2008/2009 (wave 3), 2011/2012 (wave 4), 2013 (wave 5), 2015 (wave 6), 2017 (wave 7), 2019/2020 (wave 8), and 2021/2022 (wave 9). The present study utilized data from wave 2 and onward as the Czech Republic joined SHARE for wave 2. Wave 3 was not used as data on cognitive functioning were not collected.

The study population consisted of community-dwelling individuals (aged at least 50+ years old) and their partners, irrespective of age, who were interviewed using computer-assisted personal interviewing (CAPI), as previously described in detail elsewhere [11]. SHARE has been repeatedly reviewed and approved by the Ethics Committee of the University of Mannheim. All participants have provided a written consent and have been informed about the storage and use of the data and their right to withdraw the consent.

### Analytical sample

**Figure 1.**
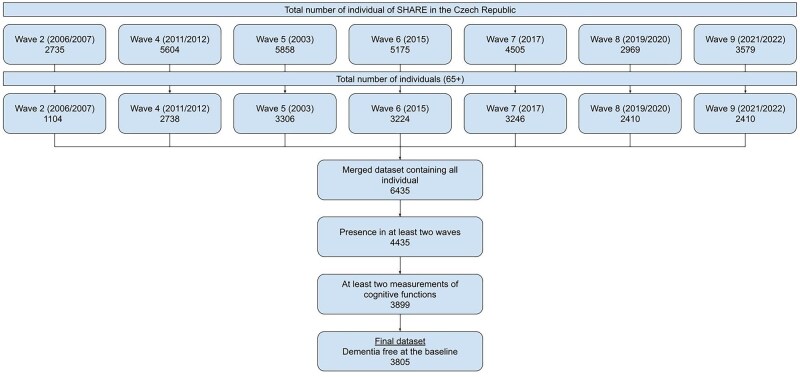
Selection of study participants.

Participants were included in the analytical sample if they fulfilled the following criteria: age 65 years or older at the time of entry into the study, participation in at least two waves of SHARE, at least two measurements of cognitive function, and no dementia at the baseline. The final study sample consisted of 3805 participants (see Fig. 1).

### Variables

#### Dementia

For the primary analysis, we classified probable dementia based on the previously adapted Lang-Weir (LW) [12] algorithmic classification. We utilized the following three measures: immediate recall, delayed recall (both from 10-words recall test), and limitations in instrumental activities of daily living (IADL). The 10-words recall test involves registering and immediate and delayed recalling a set of 10 words [13]. Limitations in IADL were measured by assessing the self-reported extent of restrictions related to practical tasks involved in daily living [14]. We used five limitations in IADL that were consistently present in all included waves: preparing meals, grocery shopping, making phone calls, taking medication, and managing money [12]. A binary variable (probable dementia vs. no dementia) was created to reflect that dementia diagnosis is a syndrome characterized by a decline in cognitive abilities severe enough to impair self-care. Specifically, first we calculated a total cognitive score by summing up the immediate and delayed recall scores (range from 0 to 20). Then, the sum of the IADL limitations (0-5) was subtracted from the total cognitive score. The LW adapted algorithm for the probable dementia status was based on the scores below global incidence based cut-off point of 6.97th percentile of the final score, as described in a previous study that also leveraged SHARE data [12]. From this point onward, we refer to the construct of “probable dementia” as “dementia.”

#### Risk factors for dementia

Possible risk factors were based on the Lancet Commission 2024 report [1] except for traumatic brain injury and air pollution, which were not collected in SHARE. Self-reported baseline information about the presence or absence of the risk factor (coded as a binary variable) was obtained during the first wave in which the individual participated. Low education was categorized as attaining levels 0-2 according to the International Standard Classification of Education [1]. Hearing loss was considered present if participants reported poor hearing. Vision problem was defined as self-reported poor near or distance vision. High blood pressure was characterized as self-reported high blood pressure diagnosed by a physician or the use of antihypertensive therapy. High cholesterol was defined in similar way by using self-reported diagnosis as well as use of drugs for high cholesterol. Diabetes mellitus was characterized by self-reported diagnosis and use of antidiabetic treatment. Alcohol use was classified as high if weekly consumption was self-reported as more than 21 units of alcohol (10 ml or 8 g pure alcohol per 1 unit) [1]. Obesity was defined as having a body mass index ≥ 30 using self-reported weight and height metrics. Participants were classified as smokers if they indicated that they had smoked daily during at some time in their life or are currently smoking. Depression was considered present if the participant had a score of four or more on the Euro-Depression scale (EURO-D) or reported the use of antidepressants medication [1]. Living alone was determined by the self-reported absence of a partner in the household. A participant was considered physically inactive if they reported that they were hardly ever, or never daily physically active.

### Statistical analysis

Participants’ characteristics at baseline are presented as frequency (n, %), mean and standard deviation (SD), or median and interquartile range (IQR), where appropriate. We conducted analyses to assess both the relative and absolute role of the risk factors in the development of dementia. To estimate the association of risk factors with incident dementia, we estimated a relative parameter—a hazard ratio (HR), with 95% confidence intervals (CI)—using Cox hazards models. Participants were censored when their status changed to dementia, in case of death, or at the end of the study period, whichever came first. We built our final model using three separate steps. First, we explored the association for each risk factor separately, adjusting only for age (in years), sex (male/female), and birth cohort (before/during/after World War II; Model 1). Second, the risk factors, which were associated with the incident dementia in Model 1 were modelled simultaneously, while adjusting for age, sex, and birth cohort (Model 2). Lastly, we added to the model also the risk factors that did not show an independent association with incident dementia to examine their role (Model 3). To test whether sex acts as an effect modifier in the association between the risk factors and dementia, we included an interaction term between the risk factors and sex (in separate models for each risk factor) and used likelihood ratio (LR) tests to assess the interaction effect. In case of evidence of interaction (*P* < .1), we additionally stratified the analysis by sex.

To assess the absolute contribution of the risk factors to the incidence of dementia, we calculated population attributable fractions (PAF). This approach follows methodology introduced previously [1]. PAF estimates the proportion of disease cases that would not occur in the population if the individual risk factor was eliminated. The formula for individual PAF is as follows: (PAF) = P_e_(RR_e_ − 1) / (1 + P_e_[RR_e_ − 1]), in which P_e_ is the prevalence of the risk factor and RR_e_ is the relative risk of disease because of that exposure. In our study, we used the HR from Model 1 instead of RR to take into account censoring. Individuals may have multiple risk factors, and as a result, individual PAFs cannot be simply summed to obtain the total PAF. Therefore, we estimated communality and calculated a weighted PAF, which takes this communality into account [1].

To assess the robustness of the results, three sensitivity analyses (SA) were performed. In SA1, missing values on risk factors were imputed using Multiple Imputation with Chained Equations. The number of imputed datasets was increased until there was a loss of efficiency of less than 5%, resulting in 10 imputed datasets. Those were analyzed separately, after which the results were pooled using Rubin’s rules. In SA2, the dementia status was constructed using an alternative LW classification for dementia based on the SHARE data [15]. In this scenario, two criteria must be separately fulfilled: summed total cognitive score lower than the prevalence-based cut-off point (4.16% for the Czech Republic [1]) and a cut-off set at 1.5 times the IQR above the third quartile for the summed IADL scores [15]. In SA3, two proxy variables were utilized to capture dementia independently of cognitive scores used in the main analysis: (i) self-reported diagnosis of dementia, senility, or Alzheimer′s disease established by a doctor and (ii) report (by respondent or proxy) of difficulty in at least one of three limitations in IADL (telephone use, taking medication, and managing finances). All analyses were performed using R version 4.3.1.

## Results

### Characteristics of participants

In total, we included 3805 participants (58.30% females; mean age at baseline 70 years; Table 1) in the analysis. During a median follow-up time of 6.50 years (IQR 4.90 years, mean 6.84 ± SD 3.37), 12.50% of them (*n* = 475) met our criteria for dementia. Participants were evenly distributed across birth cohorts. The most prevalent risk factors were high blood pressure (60%), followed by low education (40%), high cholesterol (33%), living alone (32%), obesity (30%), smoking (30%), depression (24%), diabetes mellitus (21%), physical inactivity (9%), vision problem (9%), alcohol use (8%), and hearing loss (4%).

**Table 1.** Baseline characteristics of participants (*n* = 3805)

Characteristic	Distribution
Female, *n* (%)	2219 (58.30)
Age, mean (SD)	69.80 (5.40)
Birth cohort, *n* (%)	
Before WWII	1127 (29.60)
During WWII	1305 (34.30)
After WWII	1373 (36.10)
Risk factors, *n* (%)	
Low education	1523 (40.00)
Obesity	1144 (30.10)
Alcohol use	312 (8.20)
Smoking	1134 (29.80)
Physical inactivity	349 (9.20)
High blood pressure	2291 (60.20)
High cholesterol	1265 (33.20)
Diabetes mellitus	805 (21.20)
Hearing loss	165 (4.30)
Vision problem	334 (8.80)
Depression	907 (23.80)
Living alone	1227 (32.20)

SD, standard deviation; WWII, World War II.

### Relative role of risk factors in the incidence of dementia

Out of the twelve risk factors, six were statistically significantly associated with dementia when adjusted for age, sex, and birth cohort. The largest HR was found for physical inactivity (HR 2.35; 95% CI 1.86-2.97), followed by low education (HR 1.92; 95% CI 1.58-2.34), diabetes mellitus (HR 1.77; 95% CI 1.43-2.20), depression (HR 1.69; 95% CI 1.38-2.07), obesity (HR 1.33; 95% CI 1.08-1.65), and living alone (HR 1.29; 95% CI 1.04-1.60; Table 2, Model 1). When all six risk factors were entered into the model simultaneously, only the associations of the first four risk factors (physical inactivity, low education, diabetes mellitus, and depression) weakened but remained statistically significant (Table 2, Model 2). When the other risk factors were also adjusted for, the same factors remained statistically significantly associated with incident dementia as in Model 2 (Model 3, Table 2).

**Table 2.** Association of studied risk factors with incident dementia corresponding population attributable fraction among older Czech adults in SHARE

	HR (95% CI)			Weighted PAF
	Model 1	Model 2	Model 3	
Low education	1.92 (1.58, 2.34)	1.72 (1.41, 2.10)	1.72 (1.41, 2.10)	11.42
Obesity	1.33 (1.08, 1.65)	1.17 (0.94, 1.45)	1.18 (0.95, 1.47)	3.84
Alcohol use	0.69 (0.43, 1.11)		0.72 (0.45, 1.16)	−1.11
Smoking	1.18 (0.95, 1.46)		1.19 (0.95, 1.48)	2.16
Physical inactivity	2.35 (1.86, 2.97)	2.11 (1.67, 2.68)	2.13 (1.67, 2.70)	4.69
High blood pressure	1.02 (0.84, 1.25)		0.93 (0.76, 1.15)	0.50
High cholesterol	1.04 (0.85, 1.28)		1.00 (0.81, 1.24)	0.56
Diabetes mellitus	1.77 (1.43, 2.2)	1.54 (1.24, 1.91)	1.53 (1.22, 1.91)	5.96
Hearing loss	1.30 (0.90, 1.89)		0.98 (0.67, 1.43)	0.54
Vision problem	1.28 (0.96, 1.70)		1.15 (0.86, 1.54)	1.02
Depression	1.69 (1.38, 2.07)	1.44 (1.17, 1.77)	1.42 (1.15, 1.75)	5.99
Living alone	1.29 (1.04, 1.60)	1.23 (1.00, 1.53)	1.23 (0.99, 1.53)	3.63

Weighted PAF is the relative contribution of each factor to the overall PAF when adjusted for communality. Overall weighted PAF = 39.18%.

Model 1: Each risk factor was entered into the model separately, adjusting for age, sex, and birth cohort.

Model 2: Risk factors that presented association with the outcome in Model 1 were entered into the model simultaneously, adjusting for age, sex, and birth cohort.

Model 3: All risk factors were entered into the model simultaneously, adjusting for age, sex, and birth cohort.

HR, hazard ratio; CI, confidence interval; PAF, population attributable fraction.

Sex was found to be an effect modifier in the association between dementia and high cholesterol (*P* = .01) and low education (*P* = .08). When stratified by sex (Table 3), low education was statistically significantly associated with an increased risk of dementia in all three models for both men and women High cholesterol seemed to have a weak, statistically nonsignificant association with incident dementia in females in all three models. In males, there was indication of statistically nonsignificant inverted association, with higher cholesterol linked to lower incidence of dementia.

**Table 3.** Association of studied risk factors with incident dementia among older Czech adults in SHARE stratified by sex

	HR (95% CI)	
	Male	Female
Model 1		
Low education	1.55 (1.13, 2.11)	2.22 (1.71, 2.88)
High cholesterol	0.74 (0.51, 1.08)	1.23 (0.96, 1.58)
Model 2		
Low education	1.55 (1.13, 2.12)	2.23 (1.72, 2.89)
High cholesterol	0.74 (0.51, 1.08)	1.24 (0.97, 1.59)
Model 3		
Low education	1.38 (1.00, 1.89)	2.00 (1.53, 2.60)
High cholesterol	0.64 (0.44, 0.94)	1.25 (0.97, 1.61)

Model 1: Each risk factor was entered into the model separately, adjusting for age and birth cohort.

Model 2: All two risk factors were entered into the model simultaneously, adjusting for age, sex, and birth cohort.

Model 3: All two risk factors were entered into the model simultaneously, adjusting also for obesity, smoking, physical inactivity, high blood pressure, diabetes mellitus, hearing loss, depression, age, and birth cohort.

HR, hazard ratio; 95% CI, 95% confidence interval.

### Absolute role of risk factors in the incidence of dementia

Table 2 also presents weighted PAF of each risk factor to the incidence of dementia. The overall weighted PAF was 39.18%, which is an estimate that accounts for the shared variance among the individual risk factors. Low education had the highest individual weighted PAF (11.42%), followed by depression (5.99%), diabetes mellitus (5.96%), physical inactivity (4.69%), obesity (3.84%), and living alone (3.63%).

### Sensitivity analysis

Results of SA 1 are largely in line with the results of the primary analysis, with the exception of living alone, which was statistically significantly associated with dementia in both Model 2 and 3, and vision problem, which showed a weak positive association with incident dementia in Model 1 (Supplementary Table S1). In SA 2 and SA3, compared to the main analysis, only physical inactivity and depression had a statistically significant association with dementia incidence in all three models.

## Discussion

In the present study, we assessed the role of 12 well-known risk factors in the development of dementia in a cohort of approximately 3800 community-dwelling older adults in the Czech Republic. Our analysis revealed that only four of the proposed risk factors, namely low education, depression, diabetes mellitus, and physical inactivity, were statistically significantly associated with the incidence of dementia. Our study suggests that almost 40% of dementia cases in the Czech Republic could be attributed to the combination of the presented risk factors which is slightly lower than the global estimate of the Lancet Commission (45%) [1].

In our study, low education had the highest PAF. The relationship between education and incidence of dementia is well known from other studies [16]. There are many possible mechanisms behind the role of education in this association, such as improvement of individual socioeconomic status, or brain related-pathways (e.g. the ability to utilize peak brain plasticity developed in the early ages, before reaching a plateau phase in the late adolescence [17]). The observed sex differences in the relationship between education level and incidence of dementia may be explained according to the theory of resource substitution [18]. Due to living in a patriarchal society such as the Czech Republic, females may traditionally have less power, authority, independence, and earnings than males. Therefore, access to education may be more important for females, thus exhibiting stronger effects on their dementia risks [19].

The second most prominent risk factor was depression. Our findings on depression are consistent with a comprehensive meta-analysis of 32 studies [20]. One potential biological explanation is that depression induces hippocampal damage via alterations in the noradrenergic system [21]. Additionally, previously proposed neuroinflammatory mechanisms, involving changes in TGF-beta1 and pro-inflammatory cytokines, may also contribute to this phenomenon [22].

The third factor of importance in our study was diabetes mellitus. There is a great body of evidence supporting the association of diabetes mellitus with incident dementia [23]. The potential direct mechanisms include vascular changes, such as atherosclerosis or hyper or hypoglycemic peeks leading to direct promotion of amyloid plaque and tau protein formation [24].

In our study, living alone was associated with the risk of dementia (PAF 9%), which is in line with the hypothesis that low levels of social activity are associated with an increased risk of developing cognitive impairment and dementia in older individuals [25].

Finally, physical inactivity had PAF of 4.69%. Previous studies confirm that physical inactivity is a risk factor for dementia [26]. Physically inactive people could be lacking the beneficial effect of exercise: vascular health promotion or enlargement of gray matter [27].

The lack of a statistically significant association between alcohol use and risk of dementia in our study is surprising and not in line with previous literature [28]. However, another systematic review capitalizing on 45 studies reported a reduced risk of dementia in case of light to moderate drinking as compared with not drinking, whereas heavy to excessive drinking did not affect the risk [29]. Another study found a U-shape relationship, suggesting that only too low or too high levels of alcohol consumption are associated with the increased risk of dementia [30]. However, there seems to be high variability in the current evidence and discrepancies in the definition of alcohol consumption as well as drinking patterns [29]. In our study, we may have captured moderate drinking with our definition.

Despite the fact that a meta-analysis showed a consistent statistically significant association between obesity and the risk of dementia [31], we did not observe this in the majority of our analyses. The biological mechanism, however, is currently unclear, as it is possibly influenced by a combination of many factors, such as diet, frailty, or late-life change in leptin levels [32]. Similarly to the other risk factors, the results could be also biased due to the relatively short follow-up period [33].

There was no statistically significant relationship between smoking and incidence of dementia in our study, even though the direction of our estimates suggests an increased risk, which is in line with previous literature [34]. The strength of the associations may have been weakened because of information bias due to imprecise formulation of the question. As participants reported if they ever smoked daily or not, we may have not been able to capture the intensity, duration, and timing of smoking adequately. Furthermore, there could be survival bias as many individuals may have died from smoking-associated causes before entering the study.

Our study results are not in line with a meta-analysis utilizing evidence from three prospective studies that found an association between hearing loss and the risk of dementia [35]. This could be explained mainly by the information bias and missing information about the extent and length of hearing loss. In addition, the prevalence of hearing loss in our study is low (4.3% vs. 32% in the Lancet Commission [1]). This could be due to a different definition of hearing loss or due to the fact that people with hearing disabilities may not have participated in the study. Even though the results of the association between the vision problem and dementia risk are not statistically significant, which could be possibly explained the same way as in the case of hearing loss, the observed direction is consistent with the current literature. One meta-analysis of 12 prospective studies observed risk ratio of 1.47 [36].

The absence of an association between high blood pressure and the risk of dementia in all analyses is not in line with current evidence [37]. However, the short follow-up in our study may be a likely explanation. It is a mid-life high blood pressure, which is consistently associated with an increased risk of a late-life dementia, but when blood pressure is measured later in life, the results are inconsistent [38]. As a consequence of neurodegenerative processes, the blood pressure may drop in the prodromal stages of dementia, bringing again the risk of possible bias due to the reverse causality, possibly explaining why we did not observe a statistically significant association.

Same mechanisms may underlie the nonsignificant association observed between dementia incidence and high cholesterol [39]. From the perspective of sex-differences, based on nonsignificant association, it seems that high cholesterol could play a role of a protective factor for men and risk factor for women. Those observations are in line with a study using data from the UK Biobank [40].

In addition to the limitations already mentioned, several more need to be stated. Dementia was not operationalized by a clinical evaluation but defined based on cognitive tests and IADL, which does not reflect a gold standard clinical and diagnostic practice, possibly leading to misclassification of dementia.

There is likely information bias present as the data on all the risk factors are self-reported Additionally, recall bias may be present for risk factors that were defined based on the self-reported use of drugs (for diabetes mellitus and high blood pressure). Another limitation could be possible bias due to the missing data, weakening the statistical power of the analysis. However, we repeated the analysis using MICE approach and came to the same conclusions. Furthermore, the exclusion of people who are admitted to a long-term care institutions from the sampling frame likely also underestimate the studied associations since they tend to have higher burden of studied risk factors as well as dementia.

Conversely, this study’s strengths are the population-based approach and inclusion of a well-characterized sample of older individuals from the Czech Republic, with cohesive data collection methodology that makes the individual waves comparable. To the best of our knowledge, this is the first study conducted in the Czech Republic and in the region of CEE that reports a specific role of risk factors in the development of dementia.

Our study shows that more systematic attention should be paid to mitigating the negative effect of risk factors on the incidence of dementia. From a healthcare provider perspective, better prevention and treatment of depression and diabetes mellitus may reduce the incidence of dementia. Prevention should focus on enhancing physical activity among the general population. Finally, from a societal perspective interventions to improve education and reduce solitary living could contribute to a reduced incidence of dementia in the Czech Republic. Future studies should replicate the findings of this study in other CEE countries and ideally should have a longer follow-up period to prevent reverse causation.

## Supplementary Material

ckaf112_Supplementary_Data

## Data Availability

The data underlying this article were provided by SHARE by permission. Data will be shared on request to the corresponding author with permission of SHARE. Key points Collectively, in the Czech Republic nearly 40% of dementia incidence in the Czech Republic could be preventable through targeted interventions on the main dementia risk factors. Four major risk factors—low education, depression, diabetes mellitus, and physical inactivity—were significantly associated with increased dementia risk. Sex was found to be an effect modifier only in the association between dementia and high cholesterol and low education.
