# Supplementary material for: Assessing modifiable risk factors for dementia in the Czech Republic: findings from the Survey of Health, Ageing and Retirement in Europe study
Source: Eur J Public Health. 2025 Jul 9;35(4):701–7. doi: 10.1093/eurpub/ckaf112 (PMC12311330; doi:10.1093/eurpub/ckaf112)
Supplement: ckaf112_Supplementary_Data [file ckaf112_supplementary_data.docx]

**Supplementary Table 1** Results of sensitivity analysis

| Sensitivity analysis 1 | | | |
| --- | --- | --- | --- |
| HR (95% CI) | | | |
|  | Model 1 | Model 2 | Model 3 |
| Low education | 1.96 (1.63; 2.37) | 1.74 (1.44; 2.11) | 1.75 (1.44; 2.12) |
| Obesity | 1.32 (1.07; 1.61) | 1.16 (0.94; 1.42) | 1.17 (0.95; 1.45) |
| Alcohol use | 0.84 (0.55; 1.27) |  | 0.84 (0.55; 1.27) |
| Smoking | 1.13 (0.92; 1.39) |  | 1.11 (0.90; 1.38) |
| Physical inactivity | 2.31 (1.82; 2.92) | 2.06 (1.63; 2.61) | 2.06 (1.62; 2.60) |
| High blood pressure | 1.00 (0.83; 1.21) |  | 0.90 (0.74; 1.09) |
| High cholesterol | 1.05 (0.87; 1.28) |  | 1.00 (0.82; 1.22) |
| Diabetes | 1.68 (1.37; 2.06) | 1.43 (1.16; 1.76) | 1.44 (1.16; 1.78) |
| Hearing loss | 1.30 (0.91; 1.85) |  | 1.00 (0.69; 1.44) |
| Vision problem | 1.32 (1.01; 1.72) | 1.19 (0.91; 1.56) | 1.18 (0.90; 1.55) |
| Depression | 1.79 (1.48; 2.17) | 1.50 (1.23; 1.82) | 1.50 (1.23; 1.83) |
| Living alone | 1.31 (1.07; 1.60) | 1.23 (1.00; 1.51) | 1.23 (1.00; 1.51) |
| Sensitivity analysis 2 | | | |
|  | Model 1 | Model 2 | Model 3 |
| Low education | 1.70 (1.09; 2.63) | 1.49 (0.95; 2.34) | 1.42 (0.90; 2.24) |
| Obesity | 1.29 (0.79; 2.10) |  | 1.17 (0.72; 1.93) |
| Alcohol use | 0.67 (0.21; 2.17) |  | 0.75 (0.23; 2.44) |
| Smoking | 0.87 (0.51; 1.47) |  | 0.83 (0.49; 1.42) |
| Physical inactivity | 3.55 (2.23; 5.65) | 3.26 (2.04; 5.23) | 3.16 (1.96; 5.10) |
| High blood pressure | 0.94 (0.60; 1.45) |  | 0.86 (0.55; 1.36) |
| High cholesterol | 1.03 (0.65; 1.63) |  | 1.04 (0.65; 1.67) |
| Diabetes | 1.59 (0.98; 2.57) |  | 1.37 (0.83; 2.26) |
| Hearing loss | 1.14 (0.52; 2.51) |  | 0.85 (0.38; 1.91) |
| Vision problem | 1.58 (0.89; 2.82) |  | 1.52 (0.84; 2.74) |
| Depression | 1.97 (1.27; 3.06) | 1.65 (1.05; 2.59) | 1.65 (1.05; 2.59) |
| Living alone | 1.19 (0.73; 1.93) |  | 1.08 (0.66; 1.79) |
| Sensitivity analysis 3 | | | |
|  | Model 1 | Model 2 | Model 3 |
| Low education | 1.31 (0.95; 1.82) |  | 1.12 (0.80; 1.58) |
| Obesity | 1.34 (0.93; 1.93) |  | 1.23 (0.85; 1.77) |
| Alcohol use | 1.17 (0.60; 2.26) |  | 1.37 (0.70; 2.68) |
| Smoking | 0.77 (0.52; 1.15) |  | 0.73 (0.49; 1.10) |
| Physical inactivity | 2.43 (1.66; 3.56) | 2.20 (1.50; 3.23) | 2.22 (1.51; 3.27) |
| High blood pressure | 1.27 (0.90; 1.79) |  | 1.14 (0.79; 1.63) |
| High cholesterol | 1.33 (0.95; 1.85) |  | 1.21 (0.85; 1.71) |
| Diabetes | 1.49 (1.03; 2.14) | 1.36 (0.94; 1.97) | 1.28 (0.87; 1.87) |
| Hearing loss | 1.53 (0.90; 2.61) |  | 1.25 (0.73; 2.15) |
| Vision problem | 1.04 (0.63; 1.71) |  | 0.98 (0.59; 1.63) |
| Depression | 2.02 (1.45; 2.82) | 1.88 (1.34; 2.63) | 1.82 (1.29; 2.57) |
| Living alone | 0.83 (0.58; 1.21) |  | 0.78 (0.53; 1.14) |
| *Note: HR, hazard ratio; 95% CI, 95% confidence interval;*  *Model 1: each risk factor was entered into the model separately, adjusting for age and birth cohort.*  *Model 2: all three risk factors were entered into the model simultaneously, adjusting for age, sex and birth cohort.*  *Model 3: all three risk factors were entered into the model simultaneously, adjusting also for obesity, smoking, physical inactivity, high blood pressure, diabetes mellitus, hearing loss, depression, age, and birth cohort.* | | | |
